# Measuring or estimating glomerular filtration rate in potential kidney donors: a comparative study

**DOI:** 10.1093/ckj/sfag250

**Published:** 2026-08-04

**Authors:** Rouvick Mariano Gama, Tayeba Roper, Nikhil Johri, Hugh Gallagher, Mysore Keshavmurthy Phanish

**Affiliations:** Renal and Transplantation Unit, St Helier Hospital, Epsom and St Helier University Hospitals NHS trust, Wrythe Lane, Carshalton, UK; Faculty of Life Sciences and Medicine, King’s College London, London, UK; Renal and Transplantation Unit, St Helier Hospital, Epsom and St Helier University Hospitals NHS trust, Wrythe Lane, Carshalton, UK; Department of Clinical Biochemistry, St Helier Hospital, Epsom and St Helier University Hospitals NHS trust, Wrythe Lane, Carshalton, UK; Renal and Transplantation Unit, St Helier Hospital, Epsom and St Helier University Hospitals NHS trust, Wrythe Lane, Carshalton, UK; Renal and Transplantation Unit, St Helier Hospital, Epsom and St Helier University Hospitals NHS trust, Wrythe Lane, Carshalton, UK; SW Thames Institute For Renal Research, St Helier Hospital, Epsom and St Helier University Hospitals NHS trust, Wrythe Lane, Carshalton, UK; City St Georges, University of London, Cranmer Terrace, London, UK

**Keywords:** creatinine, cystatin-C, estimated GFR, kidney donor, measured GFR

## Abstract

**Background and hypothesis:**

Accurate assessment of glomerular filtration rate (GFR) is essential during evaluation of prospective kidney donors. While estimated GFR (eGFR) equations are widely used in clinical practice, evidence regarding their performance in individuals with preserved renal function remains limited. We evaluated the agreement of creatinine, cystatin-C, and combined biomarker eGFR equations against measured GFR (mGFR) calculated using iohexol plasma clearance in healthy prospective kidney donors.

**Methods:**

We conducted a retrospective observational study of adults undergoing kidney donor assessment at a tertiary UK centre between 2015 and 2018. Serum creatinine and cystatin-C were compared with iohexol plasma clearance. Ten validated eGFR equations were evaluated. Agreement was assessed using Lin’s concordance correlation coefficient, bias, P10, P30, and Bland–Altman analyses. Sensitivity and specificity were calculated for each eGFR equation against British Transplant Society age- and sex-specific mGFR donor suitability thresholds.

**Results:**

One hundred seven participants were included; 50.5% were females, mean age was 48.5 ± 11.8 years and mean mGFR was 86.5 ± 14.6 ml/min/1.73 m^2^. The Lund–Malmo revised equation (LMR) had the most favourable agreement metrics, followed by European Kidney Function Consortium (EKFC). LMR had the highest correlation coefficient (*r* = 0.70; 95% confidence intervals (CI): 0.58 to 0.78), smallest bias (−1.3 ± 10.9 ml/min/1.73 m^2^), highest P30 (97.2%; 95% CI: 94.1–100), and highest P10 (57.0%; 95% CI 47.6–66.4). P30 exceeded 80% across most equations, whereas P10 was lower, ranging from 29.0% to 57.0%. Sensitivity for identifying donor suitability thresholds was generally high, ranging from 75.6% to 98.7%, whereas specificity was lower, ranging from 17.2% to 58.6%.

**Conclusions:**

In healthy kidney donors with preserved kidney function, creatinine-based equations, particularly LMR and EKFC, demonstrated improved agreement with iohexol plasma clearance than cystatin-C-based approaches. Despite acceptable population-level performance, low P10 and substantial variability support continued use of mGFR during evaluation of prospective kidney donors.

KEY LEARNING POINTS **What was known:**

- Estimated glomerular filtration rate (GFR) equations are widely used in clinical practice, but their performance varies according to population characteristics including age, ethnicity, body composition, and kidney function. Evidence in individuals with preserved kidney function remains limited.
- Prospective studies in predominantly White British populations with chronic kidney disease (CKD) stage 3 demonstrated good performance of creatinine and cystatin-C based equations, including Chronic Kidney Disease Epidemiology Collaboration (CKD-EPI) and European Kidney Function Consortium (EKFC), with high P30 accuracy.
- Accurate kidney function assessment is essential during living kidney donor evaluation; however, whether estimated GFR (eGFR) equations provide sufficient precision to replace measured GFR (mGFR) in healthy donor populations has remained uncertain.

**This study adds:**

- In a cohort of healthy UK kidney donors with preserved kidney function, creatinine-based equations demonstrated improved agreement with mGFR calculated using iohexol plasma clearance than cystatin-C or combined cystatin-C–creatinine biomarker equations.
- The Lund–Malmo Revised and EKFC equations demonstrated smaller bias and greater accuracy than CKD-EPI equations, suggesting equation performance may differ substantially at higher GFR ranges.
- Although P30 exceeded recommended thresholds across most equations, P10 remained low and specificity for donor suitability thresholds was poor, highlighting limitations in precision and accuracy for individual-level assessment.

**Potential impact:**

- For routine clinical decision-making and medication prescribing, eGFR performance in individuals with preserved kidney function may provide reassurance that commonly used equations remain broadly suitable at population level.
- These findings support increasing interest in European cohort-derived equations, particularly EKFC, in populations with preserved kidney function and may inform future discussions regarding equation selection.
- Despite acceptable population-level performance, measured GFR should remain the preferred approach for living kidney donor assessment because imprecision may result in inappropriate donor classification.

## INTRODUCTION

Living donor kidney transplantation (LD-KT) accounts for ∼37% of all kidney transplants worldwide and continues to increase in incidence, with a reported 2% rise in 2024 [1]. Outcomes for living donor kidney transplant recipients are generally favourable compared to deceased donor recipients, with only a small increased risk of end-stage kidney disease or major adverse outcomes compared to the general population [2–4]. Therefore, thorough assessment of prospective kidney donors is essential to minimize additional long-term risks. Central to this assessment is an accurate assessment of kidney function, to help determine donor suitability. In the United Kingdom (UK), this is directed by British Transplantation Society (BTS) guidelines, which include age- and sex-specific thresholds for glomerular filtration rate (GFR) [5].

The reference standard for assessing kidney function is measured GFR (mGFR), determined using serial sampling of exogenous filtration markers, such as iohexol or radio-labelled isotopes [6]. This may be performed using measurement of plasma clearance or urinary clearance with the gold standard reference being urinary clearance of inulin, although this is rarely used in clinical practice and far more infrequently now in research [7, 8]. However, these methods are resource-intensive, costly, and time consuming which limits their clinical utility to specific indications including chemotherapy dosing or kidney donor evaluation. In routine clinical practice estimated GFR (eGFR) is widely used instead, calculated using demographic variables (age and sex assigned at birth) and endogenous biomarkers, such as serum creatinine or cystatin-C [7].

Several eGFR equations are currently used to estimate kidney function, including the Chronic Kidney Disease Epidemiology Collaboration (CKD-EPI) 2009 and 2021, Modification of Diet in Renal Disease (MDRD), and European Kidney Function Consortium (EKFC) equations [9–11]. However, equation performance varies across populations, likely due to differences in characteristics such as age, sex, ethnicity, diet, genetic variation, body composition, and those with preserved GFR [12–16].

In the UK, the eGFR-C study demonstrated excellent performance of both CKD-EPI and EKFC creatinine and cystatin-C based equations in a predominantly White British adult cohort with chronic kidney disease (CKD) stage 3 (*n* = 1167), with 30% accuracy (P30) ranging from 88.0% to 94.9% [17, 18]. However, evidence in populations with near-normal kidney function is more limited. This is an important gap, as individuals with preserved kidney function represent the majority of the population and include prospective kidney donors. Furthermore, accurate GFR estimation at higher levels is increasingly relevant given the use of eGFR thresholds to guide initiation and dosing of renally excreted medications.

With the increasing incidence of LD-KT, there is also a need to evaluate whether eGFR equations can provide sufficiently accurate estimates of kidney function in healthy donor populations, potentially reducing reliance on more complicated and time consuming mGFR testing. This may be particularly valuable in settings where access to mGFR testing is limited.

Therefore, the aim of this study was to evaluate the performance of validated creatinine and/or cystatin-C-based eGFR equations compared to mGFR calculated using iohexol plasma clearance in a healthy prospective kidney donor cohort in the UK.

## MATERIALS AND METHODS

We conducted a retrospective observational cohort study between 2015 and 2018 (inclusive) at a single tertiary centre in South-West London. Adults undergoing evaluation for kidney donation were eligible for inclusion. Participants were excluded if they had incomplete data for key study variables.

Baseline demographic and clinical characteristics including age, sex assigned at birth, height, weight, and ethnicity were collected for all participants. Serum samples were collected for creatinine, cystatin-C, and iohexol plasma clearance at baseline (part of routine donor work-up at our centre). All analytes were measured using methodologies accredited to UKAS ISO (United Kingdom Accreditation Service International Organisation for Standardisation) 15189 laboratory standards. Serum creatinine and cystatin-C were measured on the Abbott Alinity platform. Serum creatinine concentration was measured using an enzymatic assay standardized to isotope dilution mass spectrometry and traceable to National Institute of Standards and Technology reference material (NIST SRM 967). Cystatin-C was measured using a particle-enhanced turbidimetric immunoassay (PETIA) aligned to the International Federation of Clinical Chemistry and Laboratory Medicine reference standard.

eGFR was calculated using creatinine and/or cystatin-C for the following equations: MDRD, CKD-EPI_Cr-2009_, CKD-EPI_Cr2021_, EKFC_Cr_, Lund–Malmo revised (LMR), CKD-EPI_CysC-2012_, EKFC_CysC_, CKD-EPI_CrCysC-2012_, CKD-EPI_CrCysC-2021_, and EKFC_CrCysC_ (Table S1). Ethnicity coefficients were not used with any equation.

mGFR was calculated using plasma clearance of iohexol (Omnipaque 300 mg/ml), which was administered intravenously as a 5 ml bolus. Subsequent blood samples were taken at 2, 3, and 4 h from the contralateral arm. Serum iohexol concentrations were measured using reverse-phase high performance liquid chromatography with ultraviolet detection. mGFR was calculated using the slope-intercept method with Brochner–Mortensen correction and adjusted for normalized body surface area (corrected mGFR; ml/min/1.73 m^2^) [19, 20].

The primary outcome was to evaluate agreement between creatinine, cystatin-C, and combined biomarkers eGFR equations compared to iohexol plasma clearance-derived mGFR. Data were presented as counts and percentages, mean ± standard deviation (SD), or median with interquartile range (IQR) as appropriate. Normality was assessed with histograms and Shapiro–Wilk test. eGFR bias was predominantly normally distributed and therefore reported as mean ± SD. Median bias with IQR was also reported as a robust summary measure, given the sample size and potential influence of skewness or outlying observations.

Agreement was evaluated between each eGFR equation and mGFR using Lin’s concordance correlation coefficient, bias (eGFR minus mGFR), precision (SD or IQR), 30% accuracy (P30), 10% accuracy (P10), and limits of agreement.

Lin’s concordance correlation coefficient was used as it provides complementary agreement analysis evaluating the strength and direction of the correlation between reference and index tests, and departure from the line of equality, thus assessing proportional and systematic bias. 95% confidence intervals (CI) were calculated for mean bias, median bias, and P10/P30, using the standard error of the mean difference, a distribution-free order-statistic method based on the binomial approximation and the normal approximation to the binomial proportion, respectively. Passing–Bablok regression and Bland–Altman plots provided graphical assessment of agreement. An exploratory linear regression analysis was performed to evaluate for residual confounding of eGFR bias by age and sex. For each equation, bias was the dependent variable with age and sex (Reference: female) as independent variables. Beta-coefficient with 95% CI, statistical significance, and *R*^2^ were reported for each model.

The secondary outcome was to evaluate the clinical impact of each eGFR equation on assessment of donor suitability. Age- and sex-specific mGFR thresholds recommended by the British Transplant Society Living Kidney Donor Guidelines 2018 were used (Table S2). The proportion of mGFR results above and below the thresholds were reported. Sensitivity and specificity (with 95% CI), positive predictive value (PPV), and negative predictive value (NPV) were calculated for each eGFR equation and expressed as percentages. Sensitivity was defined as the proportion of participants with mGFR above the BTS age- and sex-specific mGFR threshold for suitability to donate, who were correctly classified as above the threshold stratified by eGFR equation. Specificity was defined as the proportion of participants below the BTS mGFR threshold who were correctly classified as below the threshold stratified by eGFR equation.

Missing data were handled as a complete case analysis for key variables, which included corrected mGFR, serum creatinine, serum cystatin-C, age, and sex assigned at birth. All analyses were performed using R Software V4.4.1.

The study obtained ethical approval from the UK Health Research Authority Research Ethics Committee (Ref: 19/YH/0195).

## RESULTS

There were 117 participants who were included in the study, of which 10 were excluded due to having incomplete datasets. There were 54 (50.5%) females. Mean age was 48.5 ± 11.5 years, body mass index (BMI) was 25.90 ± 3.27 kg/m^2^, and the majority of participants were from white ethnic backgrounds (*n* = 104; 97.2%). Mean corrected mGFR was 86.5 ± 14.6 ml/min/1.73 m^2^, mean creatinine concentration was 74 ± 15 μmol/l, and median cystatin-C concentration was 0.85 mg/l (IQR 0.77 to 0.94). Mean eGFR for each equation and demographic characteristics are summarized in Table 1. There were 13 individuals who had hypertension (blood pressure was controlled with a maximum of two medications as per BTS guidelines), 13 individuals who were current or ex-smokers, two individuals with a history of gestational diabetes, and eight individuals with other comorbidities, which are summarized in Table S3.

**Table 1:** Summary of baseline demographic and clinical characteristics.

Variable	Unit
Number of participants, N (%)	107 (100)
Sex, N (%): male female	53 (49.5) 54 (50.5)
Age ± SD, years	48.5 ± 11.8
Ethnicity, N (%):	
White	104 (97.2)
black	3 (2.8)
BMI ± SD, kg/m^2^	25.90 ± 3.27
BSA ± SD, m^2^	1.83 ± 0.21
Serum creatinine concentration ± SD, μmol/l	73.9 ± 14.5
Serum cystatin-C concentration (IQR), mg/l	0.85 (0.77 to 0.94)
Corrected mGFR ± SD, ml/min/1.73 m^2^	86.5 ± 14.6
Creatinine-based eGFR ± SD ml/min/1.73 m^2^:	
MDRD	88.6 ± 19.5
CKD-EPI_Cr-2009_	94.5 ± 15.9
CKD-EPI_Cr-2021_	98.0 ± 15.3
EKFC	90.6 ± 15.1
LMR	85.2 ± 13.4
Cystatin-C-based eGFR ± SD ml/min/1.73 m^2^:	
CKD-EPI_CysC-2012_	96.5 ± 19.3
EKFC_CysC_	92.8 ± 15.4
Combined creatinine–cystatin-C eGFR ± SD ml/min/1.73 m^2^:	
CKD-EPI_CrCysC-2012_	96.3 ± 16.4
CKD-EPI_CrCysC-2021_	100.4 ± 16.1
EKFC_CrCysC_	91.7 ± 13.7

Abbreviations: BSA = Body surface area (DuBois equation).

**Figure 1:**
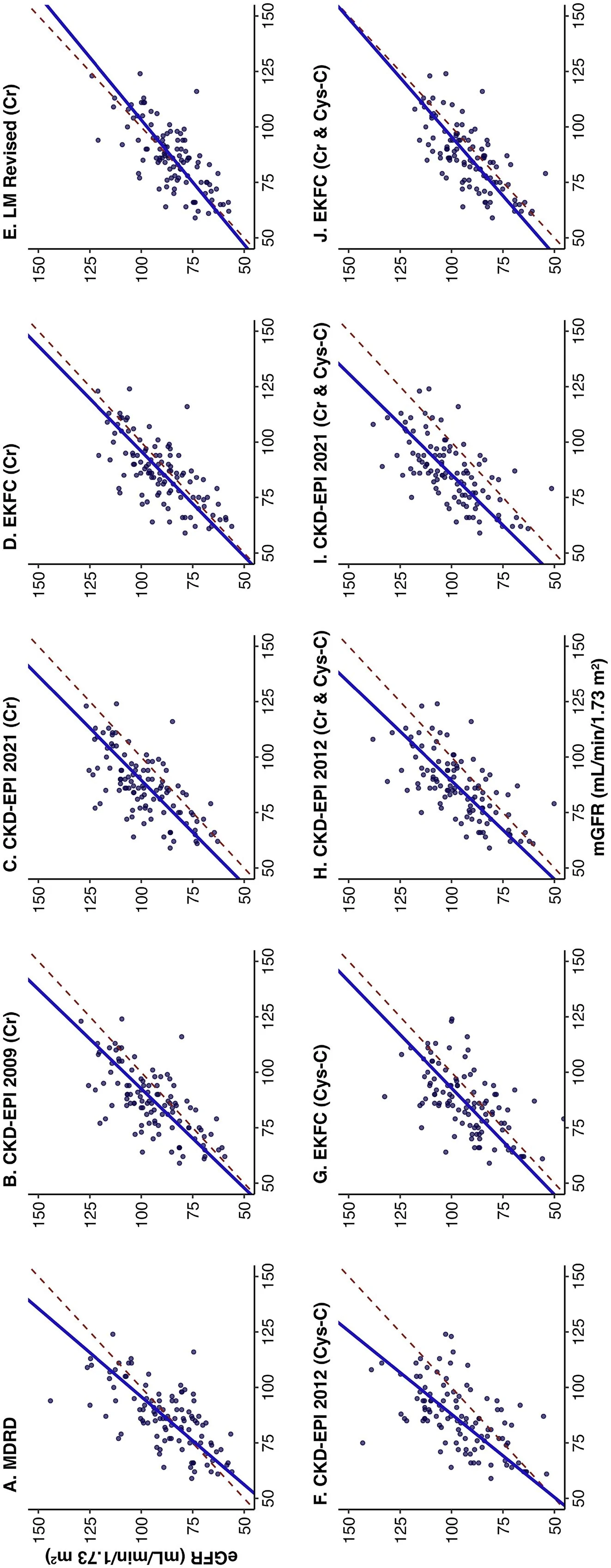
Passing–Bablok regression analyses stratified by eGFR equation against mGFR.

**Figure 2:**
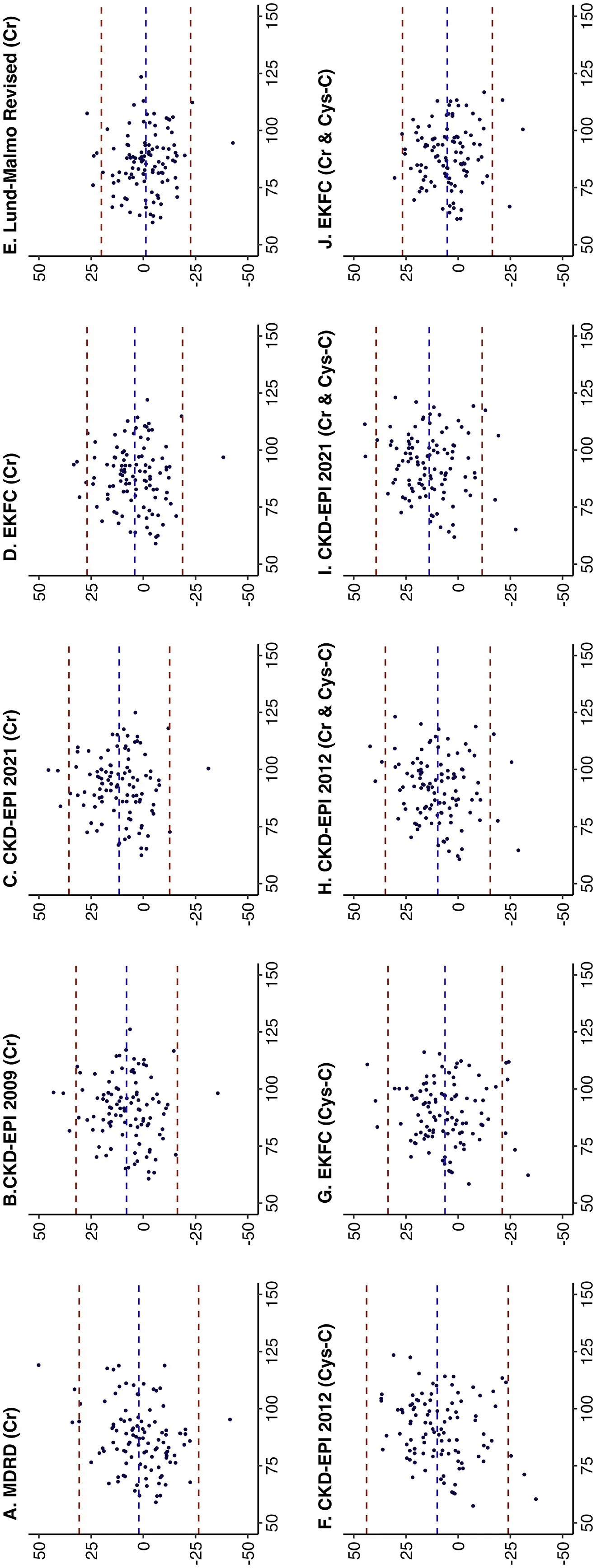
Bland–Altman plots illustrating agreement between mGFR and each eGFR equation.

Overall, LMR had the best agreement with the smallest bias, highest accuracy, and narrowest limits of agreement (Fig. 1). Across most equations, mean bias was positive, indicating overestimation of GFR, and ranged from 2.1 ± 14.6 ml/min/1.73 m² for the MDRD equation to 13.9 ± 13.0 ml/min/1.73 m² for the CKD-EPI_CrCysC-2021_. The LMR equation demonstrated the smallest mean bias (−1.3 ± 10.9 ml/min/1.73 m²). Bland–Altman plots are illustrated in Fig. 2.

**Figure 3:**
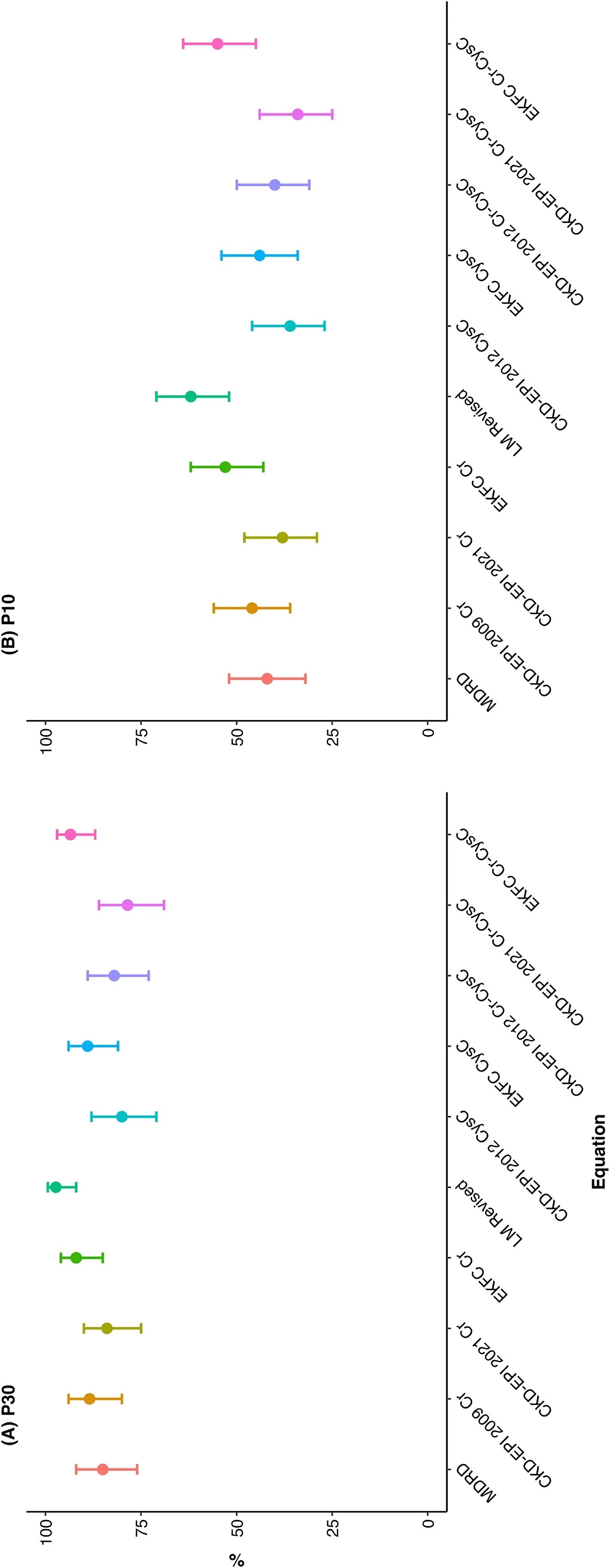
P30 and P10 with 95% confidence intervals for each eGFR equation against mGFR.

Accuracy within 30% of mGFR (P30) was high across all eGFR equations ranging from 78.5% (95% CI: 70.7–86.3) with the CKD-EPI_CrCysC2021_ to 97.2% (95% CI: 94.1–100) with LMR (Fig. 3). In contrast, P10 was substantially lower ranging from 29.0% (95% CI: 20.4–37.6) with the CKD-EPI_CrCysC2021_ to 57% (95% CI: 47.6–66.4) with LMR. Agreement metrics stratified by eGFR equation are summarized in Table 2.

**Table 2:** Bias, precision, root mean square error, accuracy and 95% limits of agreement for each eGFR equation compared to mGFR.

Equation	Mean bias [95% CI]	SD	Median bias [95% CI]	IQR	RMSE	P30 [95% CI]	P10 [95% CI]	95% Limits of agreement
MDRD	2.1 [−0.7 to 4.9]	14.6	1.8 [−1.7 to 4.6]	−7.8 to 9.3	14.7	91.6 [86.3 to 96.9]	45.8 [36.4 to 55.2]	−26.5 to 30.7
CKD-EPI_Cr-2009_	8.0 [5.6 to 10.4]	12.4	7.1 [3.6 to 10.7]	−0.1 to 15.8	14.7	87.9 [81.7 to 94.0]	44.9 [35.4 to 54.3]	−16.3 to 32.3
CKD-EPI_Cr-2021_	11.5 [9.2 to 13.9]	12.3	9.9 [7.5 to 14.7]	3.1 to 19.9	16.8	83.2 [76.1 to 90.3]	43.9 [34.5 to 53.3]	−12.7 to 35.7
EKFC	4.1 [1.8 to 6.3]	11.7	3.5 [−0.6 to 7.5]	−3.2 to 10.4	12.3	91.6 [86.3 to 96.9]	52.3 [42.9 to 61.8]	−18.8 to 26.9
LM Revised	−1.3 [−3.4 to 0.8]	10.9	−1.3 [−4.5 to 0.0]	−8.4 to 5.9	10.9	97.2 [94.1 to 100.0]	57.0 [47.6 to 66.4]	−22.6 to 20.0
CKD-EPI_CysC-2012_	10.0 [6.6 to 13.3]	17.3	10.3 [6.5 to 14.7]	0.6 to 20.9	19.9	80.4 [72.9 to 87.9]	31.8 [23.0 to 40.6]	−24.0 to 43.9
EKFC_CysC_	6.3 [3.6 to 9.0]	14.0	6.5 [3.1 to 10.8]	−1.3 to 15.3	15.3	88.8 [82.8 to 94.8]	41.1 [31.8 to 50.4]	−21.1 to 33.7
CKD-EPI_CrCysC-2012_	9.8 [7.3 to 12.2]	12.8	9.5 [7.0 to 13.5]	1.7 to 18.0	16.1	85.1 [78.3 to 91.8]	39.3 [30.0 to 48.5]	−15.4 to 34.9
CKD-EPI_CrCysC-2021_	13.9 [11.4 to 16.4]	13.0	14.0 [11.7 to 18.2]	6.2 to 22.8	19.0	78.5 [70.7 to 86.3]	29.0 [20.4 to 37.6]	−11.6 to 39.3
EKFC_CrCysC_	5.2 [3.1 to 7.3]	11.0	4.5 [2.5 to 6.3]	−1.3 to 12.8	12.1	93.5 [88.8 to 98.1]	54.2 [44.8 to 63.7]	−16.4 to 26.7

### Creatinine-based eGFR equations

The LMR had the highest performance with a correlation coefficient of 0.70 (95% CI: 0.58–0.78), followed by EKFC (0.67; 95% CI: 0.55–0.76). The CKD-EPI_Cr-2021_ had the lowest correlation with mGFR at 0.51 (95% CI: 0.39–0.61). Concordance correlation coefficients are summarized in Table 3. LMR had negative systematic bias of −1.3 ± 10.9 ml/min/1.73 m^2^, whereas the other equations all overestimated mGFR (i.e. positive bias) with bias ranging from 2.1 ± 14.6 ml/min/1.73 m^2^ (MDRD) to 11.5 ± 12.3 ml/min/1.73 m^2^ (CKD-EPI_Cr-2021_). However, there was substantial imprecision with standard deviations ranging from 10.9 to 14.6 ml/min/1.73 m^2^. P30 was greater than 80% for all equations ranging from 83.2% (95% CI: 76.1–90.3) with CKD-EPI_Cr-2021_ to 97.2% (95% CI: 94.1–100) with LMR. For P10, there was a similar trend with LMR having the greatest accuracy (57.0%; 95% CI: 47.6–66.4) and CKD-EPI_Cr-2021_ having the lowest (43.9%; 95% CI: 34.5–53.3), however, all results were substantially lower than P30 (Table 2).

**Table 3:** Lin’s concordance correlation coefficient, with 95% confidence intervals, systematic bias, proportional bias (scalar shift) and the bias correction factor.

Equation	Correlation coefficient	Systematic bias	Proportional bias	Bias correction factor
MDRD	0.64 [0.52 to 0.73]	1.33	0.13	0.95
CKD-EPI_Cr-2009_	0.59 [0.47 to 0.69]	1.09	0.53	0.88
CKD-EPI_Cr-2021_	0.51 [0.39 to 0.61]	1.05	0.77	0.77
EKFC	0.67 [0.55 to 0.76]	1.03	0.28	0.96
LMR	0.70 [0.58 to 0.78]	0.92	−0.09	0.99
CKD-EPI_CysC-2012_	0.42 [0.28 to 0.54]	1.32	0.59	0.82
EKFC_CysC_	0.52 [0.38 to 0.64]	1.05	0.42	0.92
CKD-EPI_CrCysC-2012_	0.55 [0.43 to 0.65]	1.12	0.63	0.83
CKD-EPI_CrCysC-2021_	0.46 [0.34 to 0.56]	1.10	0.91	0.71
EKFC_CrCysC_	0.65 [0.54 to 0.75]	0.94	0.37	0.93

### Cystatin-C-based eGFR equations

Overall, cystatin-C-based eGFR equations had weaker correlations with mGFR compared to creatinine-based equations, ranging from 0.42 (95% CI: 0.28–0.54) with CKD-EPI_CysC-2012_ to 0.52 (95% CI: 0.38–0.64) with EKFC_CysC_. All cystatin-C-based equations demonstrated positive systematic bias, indicating overestimation of mGFR, ranging from 6.3 ± 14.0 ml/min/1.73 m² (EKFC_CysC_) to 10.0 ± 17.3 ml/min/1.73 m² (CKD-EPI_CysC-2012_). Similar to creatinine-based equations, there was substantial imprecision, with standard deviations ranging from 14.0 to 17.3 ml/min/1.73 m². P30 remained good, ranging from 80.4% (95% CI: 72.9–87.9) for CKD-EPI_CysC-2012_ to 88.8% (95% CI: 82.8–94.8) for EKFC_CysC_. For P10, accuracy was lower, ranging from 31.8% (95% CI: 23.0–40.6) for CKD-EPI_CysC-2012_ to 41.1% (95% CI: 31.8–50.4) for EKFC_CysC_, with all values substantially lower than the corresponding P30.

### Combined creatinine and cystatin-C eGFR equations

Correlation with mGFR was variable, ranging from 0.46 (95% CI: 0.34–0.56) with the CKD-EPI_CrCysC-2021_ to 0.65 (95% CI: 0.54–0.75) with EKFC_CrCysC_. All equations overestimated mGFR ranging from 5.2 ± 11.0 ml/min/1.73 m² (EKFC_CrCysC_) to 13.9 ± 13.0 ml/min/1.73 m² (CKD-EPI_CrCysC-2021_). Precision was similar to creatinine-based equations ranging from 11.0 to 13.0 ml/min/1.73 m². P30 ranged from 78.5% (95% CI: 70.7–86.3) for CKD-EPI_CrCysC-2021_ to 93.5% (95% CI: 88.8–98.1) for EKFC_CrCysC_. For P10, accuracy ranged from 29.0% (95% CI: 20.4–37.6) for CKD-EPI_CrCysC-2021_ to 54.2% (95% CI: 44.8–63.7) for EKFC_CrCysC_, with all values substantially lower than the corresponding P30.

### Exploratory linear regression analyses

Exploratory linear regression demonstrated that age was a significant predictor of residual bias for CKD-EPI_Cr-2009_ (*β* = −0.30, 95% CI: −0.49 to−0.09, *P* = 0.004), CKD-EPI_Cr-2021_ (*β* = −0.24, 95% CI: −0.45 to −0.04, *P *= 0.017), EKFC (*β* = −0.27, 95% CI: −0.46 to −0.08, *P *= 0.006), and EKFC_CrCysC_ (*β* = −0.21, 95% CI: −0.39 to −0.03, *P *= 0.020), suggesting these equations overestimate mGFR to a greater degree in younger donors in this cohort. Age was not a significant predictor of bias for MDRD, LMR, or cystatin-C-based equations. Sex was not a significant predictor of residual bias for any equation. Results are summarized in Table 4.

**Table 4:** Linear regression analyses of eGFR equation bias against age and sex.

Equation	Age *β* (95% CI]	Age *p*-value	Sex *β* (95% CI]	Sex *p*-value	*R*²
MDRD	−0.06 [−0.31 to 0.18]	0.606	0.99 [−4.72 to 6.69]	0.733	0.004
CKD-EPI_Cr-2009_	−0.30 [−0.49 to −0.09]	0.004**	−2.13 [−6.78 to 2.52]	0.367	0.079
CKD-EPI_Cr-2021_	−0.24 [−0.45 to −0.04]	0.017*	−1.83 [−6.51 to 2.86]	0.442	0.055
EKFC	−0.27 [−0.46 to −0.08]	0.006**	−1.21 [−5.61 to 3.19]	0.587	0.071
LM Revised	−0.10 [−0.28 to 0.08]	0.264	−3.87 [−8.04 to 0.30]	0.069	0.038
CKD-EPI_CysC-2012_	−0.10 [−0.38 to 0.18]	0.494	1.00 [−5.75 to 7.75]	0.769	0.006
EKFC_CysC_	−0.16 [−0.39 to 0.07]	0.170	0.60 [−4.81 to 6.01]	0.827	0.020
CKD-EPI_CrCysC-2012_	−0.20 [−0.41 to 0.01]	0.065	−1.05 [−5.99 to 3.89]	0.675	0.033
CKD-EPI_CrCysC-2021_	−0.11 [−0.32 to 0.10]	0.319	−0.64 [−5.69 to 4.41]	0.802	0.010
EKFC_CrCysC_	−0.21 [−0.39 to −0.03]	0.020*	−0.31 (−4.68 to 4.07]	0.885	0.052

*β* = regression coefficient (ml/min/1.73 m² per year for age; ml/min/1.73 m² for sex). 95% CI = 95% confidence interval. *R*² = coefficient of determination.

*p*-value: *<0.05; **<0.01; ***<0.001.

Further sensitivity analyses were performed to evaluate differences between sex and individuals with comorbidity. Females had increased positive bias compared to males, except with LMR, however, the 95% CI overlap and significance was not evaluated (Table S4). People with comorbidity had lower bias compared to people without any comorbidity. This was consistent against most eGFR equations, although significance was not evaluated. However, there was no observable difference in accuracy (P30 and P10). Results are described in Table S5.

### Sensitivity and specificity for mGFR age- and sex-specific thresholds

Sensitivity for correctly identifying suitable mGFR results ranged from 75.6% (95% CI: 65.1–83.8) with MDRD to 98.7% (95% CI: 93.1–99.8) with the CKD-EPI_Cr-2021_ equation. Sensitivity was similar across most creatinine, cystatin-C, and combined equations although it was the highest with the CKD-EPI equations.

Specificity was low ranging from 17.2% (95% CI: 7.6–34.5) with CKD-EPI_CrCysC-2021_ to 58.6% (95% CI: 40.7–74.5) with MDRD and LMR. Specificity was lower across cystatin-C and combined equations, although the 95% CI overlapped suggesting this was not significant.

Translating this into clinical practice, CKD-EPI_Cr-2021_ identified the highest number of suitable donors with 77 true positives and only 1 false negative, however it also had only 9 true negatives with 20 false positives. In contrast, MDRD and LMR had fewer false positives, with 12 false positives each, and the highest number of true negatives, with 17 true negatives each; however, this occurred at the expense of more false negatives, with 19 for MDRD and 15 for LMR. Among cystatin-C-based equations, CKD-EPI_CysC-2012_ and EKFC_CysC_ showed identical classification counts, with 69 true positives, 7 true negatives, 22 false positives, and 9 false negatives. For combined creatinine–cystatin-C equations, false positives ranged from 22 to 24 and true negatives from 5 to 7, indicating limited specificity across combined biomarker approaches. Results are summarized with PPV, NPV, true positives (TP), false positives (FP), true negatives (TN), and false negatives (FN) in Table 5.

**Table 5:** Sensitivity and specificity of the age- and sex-specific mGFR thresholds for each eGFR equation.

Equation	Sensitivity, % [95% CI]	Specificity, % [95% CI]	PPV, %	NPV, %	TP, *n*	TN, *n*	FP, *n*	FN, *n*
MDRD	75.6 [65.1 to 83.8]	58.6 [40.7 to 74.5]	83.1	47.2	59	17	12	19
CKD-EPI_Cr-2009_	96.2 [89.3 to 98.7]	34.5 [19.9 to 52.7]	79.8	76.9	75	10	19	3
CKD-EPI_Cr-2021_	98.7 [93.1 to 99.8]	31.0 [17.3 to 49.2]	79.4	90.0	77	9	20	1
EKFC	89.7 [81.0 to 94.7]	44.8 [28.4 to 62.5]	81.4	61.9	70	13	16	8
LM Revised	80.8 [70.7 to 88.0]	58.6 [40.7 to 74.5]	84.0	53.1	63	17	12	15
CKD-EPI_CysC-2012_	88.5 [79.5 to 93.8]	24.1 [12.2 to 42.1]	75.8	43.8	69	7	22	9
EKFC_CysC_	88.5 [79.5 to 93.8]	24.1 [12.2 to 42.1]	75.8	43.8	69	7	22	9
CKD-EPI_CrCysC-2012_	94.9 [87.5 to 98.0]	20.7 [9.8 to 38.4]	76.3	60.0	74	6	23	4
CKD-EPI_CrCysC-2021_	97.4 [91.1 to 99.3]	17.2 [7.6 to 34.5]	76.0	71.4	76	5	24	2
EKFC_CrCysC_	94.9 [87.5 to 98.0]	24.1 [12.2 to 42.1]	77.1	63.6	74	7	22	4

TP = True positive, where GFR and mGFR both above the BTS threshold; TN = True Negative, where eGFR and mGFR both below the BTS threshold; FP = False positive, where eGFR above but mGFR below the BTS threshold; FN = False negative, where eGFR below but mGFR above the BTS threshold.

## DISCUSSION

### Main findings

This observational study of healthy kidney donors in a predominantly White British population demonstrated that creatinine-based eGFR equations had improved agreement with iohexol plasma clearance compared with cystatin-C or combined creatinine–cystatin-C-based equations. Among all eGFR equations evaluated, the LMR had the smallest bias and greatest accuracy, closely followed by the EKFC equation. Passing–Bablok analyses demonstrated evidence of proportional bias across most equations, particularly CKD-EPI and cystatin-C-based equations, whereas LMR and EKFC showed regression lines more closely aligned with the line of identity, in keeping with this finding.

Kidney Disease: Improving Global Outcomes (KDIGO) CKD 2024 guidelines recommend that P30 should be >80% in clinical practice, with >90% defined as optimal. In our study, P30 was above 80% in all equations except for the CKD-EPI_CrCysC-2021_ equations and was above 90% with the MDRD, LMR, and EKFC (creatinine only and combined) equations highlighting that these equations were suitable for regular clinical practice for people with preserved kidney function. However, P10 was substantially lower across all eGFR equations, which highlights a limitation of eGFR in the assessment of renal function in kidney donors. In addition, the magnitude of agreement remained moderate at best (concordance correlation coefficient 0.42 to 0.70), highlighting that even the most favourable performing equations had limitations in utility for those with preserved kidney function and did not demonstrate sufficiently strong agreement with iohexol plasma clearance for individual-level donor assessment.

### Performance of creatinine-based eGFR equations

In this White British cohort with preserved kidney function, the LMR and EKFC equations demonstrated smaller bias and higher accuracy compared to the CKD-EPI equations, which are typically used in clinical practice. These findings are supported by previous studies in European populations when stratified for higher GFR thresholds. Pottel *et al*. reported a bias of 0.6 ml/min/1.73 m^2^ and accuracy of 91.1% with the EKFC equation compared to 8.7 ml/min/1.73 m^2^ and 84.8%, respectively, in 972 adults aged between 18 and 40 years old [21]. Similarly, Nyman *et al*., evaluated eGFR performance in 3495 Swedish individuals and identified that in the subgroup with mGFR between 60 and 89 ml/min/1.73 m^2^ bias was 1.9% with LMR compared to 13.1% with CKD-EPI_Cr-2009_ [22].

In contrast, the eGFR-C study demonstrated that in a predominantly White British population with CKD stage 3, accuracy was higher with the CKD-EPI_Cr-2009_ equation and that equations typically had negative bias (i.e. underestimation) compared to mGFR [17, 18]. These findings differ to our cohort, with positive bias seen in all equations with the exception of LMR, although P30 (accuracy) remained comparable between both studies. This disparity is likely related to difference in population characteristics between the two studies. In particular, our cohort had a higher mean mGFR, which is likely to be better represented by the EKFC and LMR equations. However, our cohort was also smaller (107 compared to 1167 participants) which may increase the risk of bias.

Of note, the exploratory linear regression models identified that age was a significant predictor of residual bias for CKD-EPI_Cr-2009,_ CKD-EPI_Cr-2021_, EKFC, and EKFC_CrCysC_, with greater overestimation observed in younger prospective donors. For creatinine-based CKD-EPI equations, this is not unexpected and has been demonstrated in other European cohorts [23]. However, the finding of residual age-related bias for EKFC is less expected, given that this equation incorporates age- and sex-specific creatinine reference values (*Q* values) specifically designed to attenuate age-related confounding. This may reflect cohort-specific characteristics, or alternatively may represent overfitting within this relatively small sample of 107 participants, and therefore these models should be interpreted with caution. Sex was not significantly associated with residual bias for any equation. Regardless, given the modest *R*² values across all models, age and sex collectively explain only a small proportion of the variance in bias, and other unmeasured factors likely contribute to residual variability.

### Cystatin-C and combined eGFR equations

Equations incorporating cystatin-C demonstrated greater positive bias compared to creatinine only EKFC and LMR equations. In addition, accuracy appeared lower for cystatin-C-only and combined CKD-EPI equations relative to creatinine only EKFC and LMR. Although formal statistical comparisons were not performed and the study was not powered to detect differences between equations, this pattern was consistent across multiple performance metrics.

These findings are surprising as typically cystatin-C based equations report lower GFR estimates compared to creatinine only equations [13, 23, 24]. However, several factors may contribute to this observation. First, the relatively young and healthy nature of this cohort, with preserved kidney function, may influence the relationship between filtration markers and measured GFR. In a large Swedish cohort (*n* = 6174), Fu *et al*. reported positive bias for CKD-EPI and EKFC cystatin-C-based equations at higher GFR levels, unlike LMR which consistently reported a small negative bias [23]. These findings would support the pattern of agreement identified in our cohort.

Analytical variability may also contribute. In our cohort, cystatin-C was measured using PETIA run on the Abbott Alinity platform. Variability between cystatin-C assays and analytical platforms has been reported and documented in UK NEQAS reports, including differences due to calibration and local assay performance. However, some studies have demonstrated positive bias in cystatin-C concentrations relative to reference standards with PETIA-based assays, which would be expected to result in lower eGFR estimates, which goes against the results reported in our study [18, 25, 26]. However, the direction and magnitude of this effect may vary depending on population characteristics and assay/analyzer and therefore this could still result in variability in equation performance, which may account for the findings in our study.

These findings suggest that both biological and analytical factors may influence the performance of cystatin-C-based equations in populations with preserved kidney function. However, further analyses in larger cohorts are required to corroborate these findings.

### Comparison to the existing literature of kidney donor GFR evaluation

When compared with similar studies evaluating eGFR performance in prospective kidney donor cohorts, one main difference in our cohort is that eGFR overestimated iohexol plasma clearance, whereas underestimation is more typically reported in other European cohorts [27, 28]. For example, van der Weijden *et al*. evaluated 486 prospective kidney donors from the Netherlands, with a mean mGFR of 94 ± 16 ml/min/1.73 m^2^ using urinary iothalamate as the mGFR reference test. In their study, creatinine-based and cystatin-C-based equations generally underestimated pre-donation mGFR, with bias ranging from −11.4 ml/min/1.73 m² for EKFC creatinine to +0.01 ml/min/1.73 m² for CKD-EPI creatinine–cystatin-C 2021. In contrast to our findings, combined creatinine–cystatin-C equations had the smallest bias and highest accuracy.

However, there are several potential explanations for these differences. First, cohort characteristics differed between studies. Our cohort was older and had a lower mean mGFR, both of which may influence equation performance and reduce generalizability across donor cohorts. Second, cystatin-C measurements differed between studies. Van der Weijden *et al*. measured cystatin-C calculated using calibrated PETIA method on different analytical platforms, which may be associated with substantial differences in eGFR performance as discussed earlier [18].

Third, the mGFR methods differed. van der Weijden *et al*. used urinary iothalamate clearance; in the USA, Lahorewala *et al*., also used urinary iothalamate clearance and reported underestimation of mGFR across eGFR equations (with and without cystatin-C) in a large cohort (*n* = 326–868). However, in our study we used plasma clearance of iohexol. Differences in mGFR methods may contribute to systematic differences in eGFR performance, as plasma clearance has been reported to lead to higher GFR values compared to urinary clearance, when compared with a single biomarker (e.g. iohexol) or when compared to the reference standard of urinary clearance of inulin [27, 29–31]. For example, Stehle *et al*., demonstrated slight overestimation of mGFR using plasma clearance of iohexol with the CKD-EPI equation and modest underestimation with EKFC [27]. This indicates that differences in mGFR methods alone would not explain the results in our cohort, however, a combination of heterogeneity in cohort characteristics, mGFR, and analyte methodology and potential unaccounted variables, such as non-GFR determinants of creatinine or cystatin-C may also play a role.

Despite these methodological and population differences, the clinical implication for evaluation of kidney donation is consistent across studies. Even when P30 is high, P10 remains modest, indicating substantial imprecision at the individual donor assessment level. Therefore, eGFR alone remains insufficient to reliably determine donor suitability.

### Implications for clinical practice and future research

Overall, systematic bias across commonly used eGFR equations was modest and P30 accuracy was above the recommended thresholds set by KDIGO, which may provide reassurance when using eGFR to guide clinical decision-making, particularly for medication prescribing based on eGFR threshold values. In this cohort with preserved kidney function, the LMR and EKFC equations demonstrated improved agreement with mGFR than CKD-EPI equations. Given that individuals with preserved kidney function represent the majority of the adult population, this raises the question of whether these European-derived equations may be preferable in clinical contexts, which is a question likely to be supported by the European Federation for Clinical Chemistry and Laboratory Medicine, who now recommend the EKFC equation in routine clinical practice [10].

The substantial imprecision was observed across all equations, translating to wide variability at the individual level. This has important implications for clinical practice, as it may limit confidence in eGFR estimates when precise assessment of kidney function is required. This is relevant in the assessment of prospective kidney donors, where small differences in GFR may have significant implications for eligibility. The combination of imprecision and relatively low P10 accuracy observed across all equations suggests that eGFR alone does not provide sufficient confidence that true GFR exceeds established thresholds for donation.

When assessing donor suitability using age- and sex-specific mGFR thresholds, correct identification of truly suitable donors was high across most equations but particularly with the CKD-EPI equations including CKD-EPI_Cr-2009_ which is currently recommended in UK clinical practice. However, this high sensitivity is likely driven by systematic overestimation of mGFR, which was most pronounced with CKD-EPI equations regardless of biomarker. This is reflected in the low specificity specifically across these equations, indicating a substantial risk of incorrectly classifying borderline or unsuitable donors as eligible. In contrast, MDRD and LMR demonstrated lower sensitivity but comparatively higher specificity (although this was still insufficient for clinical practice), suggesting an improvement in being able to flag donors for further evaluation, rather than incorrectly accepting them. The low specificity is concerning in this cohort as it suggests that there is a substantial risk in clinical practice of classifying ineligible prospective kidney donors as eligible kidney donors, which would put individuals at risk of long-term renal consequences.

Cystatin-C and combined biomarker equations did not demonstrate any improvement with very low specificity, highlighting that in this type of cohort, cystatin-C may have limited value and may be influenced by local assay performance to limit utility. Further work is warranted in large and diverse cohorts of individuals with preserved kidney function to evaluate the impact on analytical methods for measuring cystatin-C on the agreement with mGFR. Overall, these findings support the continued use of mGFR in the evaluation of living kidney donors, as the risk of error remains inappropriately high regardless of eGFR equation and/or endogenous biomarker.

### Strengths and limitations

Strengths of this study include same-day eGFR and mGFR testing, the inclusion of cystatin-C as well as serum creatinine, and the calculation of mGFR using plasma clearance of iohexol, which is a well-recognized reference standard. In addition, the cohort was made of up prospective kidney donors, which provides some generalizability toward the wider White British healthy population, with analyses including the main validated eGFR equations used worldwide.

Limitations include the sample size. Although there are over 100 paired observations, which meets the Clinical and Laboratory Standards Institute criteria for diagnostic agreement analyses, the sample size was not powered to assess for significant differences between bias and accuracy and therefore, this analysis and conclusions drawn remain exploratory. A regression analysis was performed to assess if there was an ongoing significant association between eGFR equation bias and age and sex. Due to sample size, these findings should be interpreted with caution as there is a risk of overfitting. In addition, further subgroup analyses of age bands were not possible due to insufficient power in age subgroups or the risk of masking heterogeneity by using large age groups.

Furthermore, the cohort lacked ethnic diversity, which limits the applicability to diverse populations. There were also limited clinical co-variates included, which limit the degree to which confounding variables may be mitigated. We acknowledge the number of samples recommended by the EKFC for iohexol plasma clearance is four samples, to allow for at least three samples in case of error for people with an eGFR > 60 ml/min/1.73 m^2^ [32]. In our protocol, we performed three timed samples at 2, 3, and 4 h post-administration of iohexol. However, this was in line with the eGFR-C study protocol. In addition, a pre-specified *R*² threshold was not routinely used to assess curve linearity during the study period. Quality assurance instead relied on visual inspection and repeat analysis where the curve appeared questionable. Consequently, objective numerical verification of curve fit was not available for all measurements, introducing the potential for inter-operator variability in curve assessment and reducing the standardization and reproducibility of the quality-assurance process. Finally, cystatin-C measurements were performed using a single assay, which, as discussed previously, may be prone to bias and impact the performance of cystatin-C-based eGFR equations.

## CONCLUSION

In a cohort of White British healthy kidney donors, creatinine-based eGFR equations demonstrated greater agreement with iohexol plasma clearance, compared to cystatin-C or combined equations. However, performance is known to be affected by choice of assay and this needs to be considered in subsequent analyses. The LMR and, to a lesser extent, EKFC had improved performance in this population with preserved kidney function compared to equations used in clinical practice, such as CKD-EPI_Cr-2009_ and MDRD.

Despite good to optimal P30 amongst most eGFR equations, there was substantial imprecision and P10 values were low with in this cohort of prospective kidney donors, which suggests performance is limited at individual level, thus supporting the continued use of mGFR in kidney donor assessment.

## Supplementary Material

sfag250_Supplemental_File

## Data Availability

A de-identified dataset may be made available upon reasonable request to the corresponding author, subject to relevant institutional approvals and data sharing agreements.
