# Supplementary material for: Measuring or estimating glomerular filtration rate in potential kidney donors: a comparative study
Source: Clin Kidney J. 2026 Aug 4;19(9):sfag250. doi: 10.1093/ckj/sfag250 (PMC13529817; doi:10.1093/ckj/sfag250)
Supplement: Supplementary file 1 [file sfag250_supplemental_file.pdf]

Estimated glomerular filtration rate performance in prospective kidney donors

Supplementary Materials

Table S1. List of eGFR equations with their respective formulae.

Abbreviation of GFR Equation	Year	Cohort Origin	Cohort Size	GFR Equation Formula
CG	1976	USA White Males	249	[(140 – age) x weight x 1.23] / Cr x 0.85 [if female]
MDRD	1999	USA Multiethnic	1628	$175 \times$ standardized $Cr^{-1.154} \times age^{-0.203} \times 1.210$ [if black] $\times 0.742$ [if female]
$CKD\text{-}EPI_{Cr\text{-}2009}$ [i]	2009	USA Multiethnic	8254	$141 \times \min(Cr/\kappa, 1)^{\alpha} \times \max(Cr/\kappa, 1)^{-1.209} \times 0.993^{Age} \times 1.159$ [if Black] x 1.018 [if female]
$CKD\text{-}EPI_{Cr\text{-}2021}$ [ii]	2021	USA Multiethnic	8254	$142 \times \min(Cr/\kappa, 1)^{\alpha} \times \max(Cr/\kappa, 1)^{-1.200} \times 0.994^{Age} \times 1.012$ [if female]
$CKD\text{-}EPI_{CysC\text{-}2012}$	2012	USA Multiethnic	5352	$133 \times \min(CysC/0.8, 1)^{-0.499} \times \max(CysC/0.8, 1)^{-1.328} \times 0.996^{Age} \times 0.932$ [if female]
$CKD\text{-}EPI_{Cr\text{-}CysC\text{-}2012}$ [iii]	2012	USA Multiethnic	5352	$135 \times \min(Cr/\kappa, 1)^{\alpha} \times \max(Cr/\kappa, 1)^{-0.601} \times \min(CysC/0.8, 1)^{-0.375} \times \max(CysC/0.8, 1)^{-0.711} \times 0.995^{Age} \times 0.969$ [if female] $\times 1.08$ [if black]
$CKD\text{-}EPI_{Cr\text{-}CysC\text{-}2021}$ [iv]	2021	USA Multiethnic	5352	$135 \times \min(Cr/\kappa, 1)^{\alpha} \times \max(Cr/\kappa, 1)^{-0.544} \times \min(CysC/0.8, 1)^{-0.323} \times \max(CysC/0.8, 1)^{-0.778} \times 0.996^{Age} \times 0.963$ [if female]
Lund-Malmo Revised (LMR) [v]	2011	White Swedish	850	eX–0·0158×Age+0·438×ln(Age)
EKFC [vi]	2021	White European	11251	Age / Cr/Q / Equation<br>2-40y / <1 / $107.3 \times (Cr/Q)^{-0.322}$<br>2-40y / ≥1 / $107.3 \times (Cr/Q)^{-1.132}$<br>>40y / <1 / $107.3 \times (Cr/Q)^{-0.322} \times 0.990^{(Age-40)}$<br>>40y / ≥1 / $107.3 \times (Cr/Q)^{-1.132} \times 0.990^{(Age-40)}$
$EKFC_{CysC}$ [vi]	2021	White European	11251	Age / CysC/Q / Equation<br>2-40y / <1 / $107.3 \times (CysC/Q)^{-0.322}$<br>2-40y / ≥1 / $107.3 \times (CysC/Q)^{-1.132}$<br>>40y / <1 / $107.3 \times (CysC/Q)^{-0.322} \times 0.990^{(Age-40)}$<br>>40y / ≥1 / $107.3 \times (CysC/Q)^{-1.132} \times 0.990^{(Age-40)}$

Abbreviations: CKD-EPI, Chronic Kidney Disease Epidemiology Collaboration; EKFC, European Kidney Function Consortium; CysC, cystatin C; Cr-CysC, creatinine-cystatin C; MDRD, Modification of Diet in Renal Disease; LMR, Lund-Malmo revised.

[i] *α = -0.329 (females) or -0.411 (males); κ = 0.7mg/dL or 62 µmol/L (females) or 0.9mg/dL or 80µmol/L (males); min = indicates the minimum of Cr/κ or 1; max = indicates the maximum of Cr/κ or 1; age = years*
[ii] *α = -0.241 (females) or -0.302 (males); κ = 0.7mg/dL or 62 µmol/L (females) or 0.9mg/dL or 80µmol/L (males)*
[iii] *α = -0.248 (females) or -0.207 (males); κ = 0.7mg/dL or 62 µmol/L (females) or 0.9mg/dL or 80µmol/L (males)*
[iv] *α = -0.219 (females) or -0.144 (males); κ = 0.7mg/dL or 62 µmol/L (females) or 0.9mg/dL or 80µmol/L (males)*
[v] *Female Cr<150: X = 2·50+0·0121 × (150– Cr)*
[v] *Female Cr ≥150: X = 2·50–0·926 × ln(Cr /150)*
[v] *Male Cr <180: X = 2·56+0·00968 × (180–Cr)*
[v] *Male Cr ≥180: X = 2·56–0·926 × ln(Cr /180)*
[vi] *Q values correspond to the median Cr or CysC values for the age- and sex-specific populations*

Table S2. Advisory threshold glomerular filtration rate levels considered acceptable for living kidney donation based on the British Transplant Society guidelines for living donor kidney transplantation

**Age (years)**	**Threshold GFR (mL/min/1.73m^2)**	
	Male	Female
20 – 29	90	90
30 – 34	80	80
35 - 39	80	80
40 – 44	80	80
45 – 49	80	80
50 – 54	80	80
55 – 59	80	75
60 – 64	76	70
65 – 69	71	64
70 – 74	67	59
75 – 79	63	54
≥ 80	58	49

Table S3. Frequency of comorbidities amongst individuals in the cohort.

**Comorbidity list**	**Count (%)**
Smoking History (Current or Ex-smoker)	13 (12.1)
Hypertension	13 (12.1)
Hypercholesterolaemia	2 (1.9)
Gestational diabetes mellitus	2 (1.9)
Other:	8 (7.5)
Basal cell carcinoma > 10 years ago (excised)	1 (0.9)
Gastroeosophageal reflux	1 (0.9)
Hiatus hernia	1 (0.9)
Hypothyroidism	1 (0.9)
Gout	2 (1.9)
Depression	1 (0.9)
Asthma	1 (0.9)

Table S4. Agreement of eGFR equations against mGFR stratified by sex.

Equation	Median bias [95% CI]	IQR	RMSE	P30 [95% CI]	P10 [95% CI]
**Male Sex**					
MDRD	0.2 [-5.6 to 5.6]	-7.5 to 9.5	15.7	88.7 [80.2 to 97.2]	52.8 [39.4 to 66.3]
CKD-EPI$_{Cr\text{-}2009}$	5.7 [2.2 to 10.2]	-0.2 to 15.5	15.2	84.9 [75.3 to 94.5]	47.2 [33.7 to 60.6]
CKD-EPI$_{Cr\text{-}2021}$	8.4 [4.9 to 13.0]	2.9 to 19.8	17.2	81.1 [70.6 to 91.7]	49.1 [35.6 to 62.5]
EKFC	2.2 [-2.0 to 8.5]	-3.0 to 10.9	13.3	86.8 [77.7 to 95.9]	52.8 [39.4 to 66.3]
LM Revised	-2.7 [-7.4 to -0.5]	-10.6 to 1.7	12.1	96.2 [91.1 to 100.0]	50.9 [37.5 to 64.4]
CKD-EPI$_{CysC\text{-}2012}$	12.1 [1.6 to 16.8]	-0.6 to 21.8	22.5	79.2 [68.3 to 90.2]	26.4 [14.6 to 38.3]
EKFC$_{CysC}$	8.8 [2.2 to 13.5]	-3.9 to 17.7	17.4	86.8 [77.7 to 95.9]	32.1 [19.5 to 44.6]
CKD-EPI$_{Cr\text{-}CysC\text{-}2012}$	12.7 [4.4 to 16.1]	-0.1 to 18.1	16.9	84.9 [75.3 to 94.5]	37.7 [24.7 to 50.8]
CKD-EPI$_{Cr\text{-}CysC\text{-}2021}$	16.8 [7.7 to 20.2]	2.9 to 23.6	19.7	75.5 [63.9 to 87.1]	34.0 [21.2 to 46.7]
EKFC$_{Cr\text{-}CysC}$	5.3 [-0.2 to 11.2]	-2.6 to 15.5	13.8	92.5 [85.3 to 99.6]	39.6 [26.5 to 52.8]
**Female Sex**					
MDRD	1.8 [-3.2 to 6.5]	-8.5 to 9.2	13.6	94.4 [88.3 to 100.0]	38.9 [25.9 to 51.9]
CKD-EPI$_{Cr\text{-}2009}$	9.2 [3.4 to 13.1]	0.2 to 16.2	14.2	90.7 [83.0 to 98.5]	42.6 [29.4 to 55.8]
CKD-EPI$_{Cr\text{-}2021}$	12.1 [7.1 to 16.9]	3.3 to 19.9	16.5	85.2 [75.7 to 94.7]	38.9 [25.9 to 51.9]
EKFC	4.4 [-0.9 to 7.9]	-4.3 to 10.0	11.2	96.3 [91.3 to 100.0]	51.9 [38.5 to 65.2]
LM Revised	-0.2 [-4.4 to 4.4]	-5.8 to 6.6	9.6	98.2 [94.6 to 100.0]	63.0 [50.1 to 75.8]
CKD-EPI$_{CysC\text{-}2012}$	8.0 [3.8 to 15.6]	2.1 to 19.7	16.9	81.5 [71.1 to 91.8]	37.0 [24.2 to 49.9]
EKFC$_{CysC}$	5.7 [1.9 to 10.8]	-1.1 to 14.1	12.9	90.7 [83.0 to 98.5]	50.0 [36.7 to 63.3]
CKD-EPI$_{Cr\text{-}CysC\text{-}2012}$	8.9 [6.6 to 12.8]	3.4 to 17.7	15.2	85.2 [75.7 to 94.7]	40.7 [27.6 to 53.9]
CKD-EPI$_{Cr\text{-}CysC\text{-}2021}$	13.3 [11.3 to 17.0]	7.4 to 22.1	18.2	81.5 [71.1 to 91.8]	24.1 [12.7 to 35.5]
EKFC$_{Cr\text{-}CysC}$	4.2 [1.8 to 5.9]	0.5 to 9.7	10.2	94.4 [88.3 to 100.0]	68.5 [56.1 to 80.9]

Table S5. Agreement of eGFR equations against mGFR stratified by individuals with comorbidity.

Equation	Median bias [95% CI]	IQR	RMSE	P30 [95% CI]	P10 [95% CI]
**Individuals with comorbidity (n = 40)**					
MDRD	−0.8 [−8.2 to 3.0]	−9.1 to 8.6	17.6	87.5 [77.2 to 97.8]	42.5 [27.2 to 57.8]
CKD-EPI$_{Cr\text{-}2009}$	3.1 [−1.7 to 9.9]	−2.7 to 12.1	13.9	90.0 [80.7 to 99.3]	55.0 [39.6 to 70.4]
CKD-EPI$_{Cr\text{-}2021}$	7.2 [1.5 to 13.0]	0.9 to 15.6	15.2	90.0 [80.7 to 99.3]	52.5 [37.0 to 68.0]
EKFC	−2.3 [−4.3 to 7.5]	−6.1 to 8.4	12.1	95.0 [88.2 to 100.0]	55.0 [39.6 to 70.4]
LM Revised	−4.5 [−8.5 to 0.0]	−9.2 to 3.3	12.4	95.0 [88.2 to 100.0]	55.0 [39.6 to 70.4]
CKD-EPI$_{CysC\text{-}2012}$	3.4 [−0.2 to 11.4]	−1.9 to 19.4	16.4	82.5 [70.7 to 94.3]	42.5 [27.2 to 57.8]
EKFC$_{CysC}$	3.0 [−2.7 to 7.4]	−4.5 to 13.2	13.7	97.5 [92.7 to 100.0]	45.0 [29.6 to 60.4]
CKD-EPI$_{Cr\text{-}CysC\text{-}2012}$	7.4 [−1.7 to 10.7]	−2.3 to 13.2	13.5	92.5 [84.3 to 100.0]	42.5 [27.2 to 57.8]
CKD-EPI$_{Cr\text{-}CysC\text{-}2021}$	12.1 [2.6 to 15.6]	2.3 to 18.6	16.0	90.0 [80.7 to 99.3]	37.5 [22.5 to 52.5]
EKFC$_{Cr\text{-}CysC}$	2.6 [−3.6 to 5.5]	−4.8 to 6.8	11.2	97.5 [92.7 to 100.0]	60.0 [44.8 to 75.2]
**Individuals with no comorbidity (n = 67)**					
MDRD	2.9 [−2.4 to 6.5]	−6.5 to 10.1	12.6	94.0 [88.4 to 99.7]	47.8 [35.8 to 59.7]
CKD-EPI$_{Cr\text{-}2009}$	9.2 [4.8 to 15.2]	2.6 to 16.7	15.2	86.6 [78.4 to 94.7]	38.8 [27.1 to 50.5]
CKD-EPI$_{Cr\text{-}2021}$	12.4 [8.4 to 17.8]	6.5 to 20.5	17.7	79.1 [69.4 to 88.8]	38.8 [27.1 to 50.5]
EKFC	4.9 [1.2 to 9.8]	−1.4 to 13.0	12.4	89.5 [82.2 to 96.9]	50.8 [38.8 to 62.7]
LM Revised	−0.5 [−3.6 to 1.7]	−6.2 to 6.9	9.9	98.5 [95.6 to 100.0]	58.2 [46.4 to 70.0]
CKD-EPI$_{CysC\text{-}2012}$	13.3 [8.0 to 16.8]	3.2 to 21.8	21.7	79.1 [69.4 to 88.8]	25.4 [14.9 to 35.8]
EKFC$_{CysC}$	10.7 [5.0 to 13.5]	1.0 to 16.1	16.1	83.6 [74.7 to 92.5]	38.8 [27.1 to 50.5]
CKD-EPI$_{Cr\text{-}CysC\text{-}2012}$	10.2 [6.3 to 12.9]	2.1 to 17.2	16.0	85.1 [76.5 to 93.6]	32.8 [21.6 to 44.1]
CKD-EPI$_{Cr\text{-}CysC\text{-}2021}$	13.5 [8.3 to 17.5]	4.7 to 19.5	17.4	80.6 [71.1 to 90.1]	37.3 [25.7 to 48.9]
EKFC$_{Cr\text{-}CysC}$	17.0 [12.1 to 21.7]	8.7 to 23.7	20.5	71.6 [60.9 to 82.4]	23.9 [13.7 to 34.1]
